# High-yield isolation of primary human hepatocytes from small liver samples

**DOI:** 10.1007/s44164-025-00097-4

**Published:** 2025-11-13

**Authors:** Thea Guy, Jia-Ling Ruan, Carl Lee, Kaitlyn Purdie, David Johnson, Alex Gordon-Weeks, Jagdeep Nanchahal

**Affiliations:** 1 Nuffield Department of Orthopaedics, Rheumatoid and Musculoskeletal Sciences, The Kennedy Institute of Rheumatology, University of Oxford, Oxford, United Kingdom; 2 Department of Oncology, University of Oxford, Oxford, United Kingdom; 3 The Nuffield Department of Surgical Sciences, University of Oxford, Oxford, United Kingdom; 4 Department of Engineering Science, University of Oxford, Oxford, United Kingdom

**Keywords:** Primary human hepatocytes, Hepatocyte isolation, Hepatocyte viability, Hepatocyte yield, Non-encapsulated liver specimen

## Abstract

**Purpose:**

Isolation of primary human hepatocytes (PHH) from liver specimens typically relies on the two-step perfusion method, which requires large samples, substantial resources, specialised expertise and suitable vessels for cannulation. Although non-perfusion methods exist, they yield low numbers of hepatocytes and inadequately assess hepatocyte purity. We compared and optimised these methods to develop an improved technique that isolates high yields of viable PHH from non-perfusable liver specimens.

**Method:**

In the optimised protocol, non-cancerous resected liver tissue (mean weight: 8.5 ± 2.0 g, SEM) was sliced into 350 μm sections using a vibratome and subjected to a two-step isolation digestion with ethylenediaminetetraacetic acid (EDTA) and collagenase. Cell yield and viability were assessed using propidium iodide staining. Cell populations were characterised by immunofluorescent imaging.

**Results:**

The optimised protocol yielded 1.17 ± 0.2 × 10^6^ viable PHH per gram of tissue, approximately 2-fold higher than other non-perfusion protocols, although lower than yields reported for perfusion protocols in the literature. Notably, our protocol achieved an average hepatocyte viability of 80 ± 4%, which surpassed the reported average for published non-perfusion methods. Staining for glycogen and albumin secretion confirmed the functional integrity of the isolated PHH. The protocol was also effective with steatotic liver tissue, yielding 1.0 ± 0.1 × 10⁶ viable PHH per gram with 83 ± 2% viability. Most liver specimens were obtained from patients who had undergone neoadjuvant chemotherapy, however, no trend related to chemotherapy treatment was observed.

**Conclusions:**

Our non-perfusion protocol permits the isolation of viable and functional PHH from a diverse range of liver samples. This advancement provides a practical alternative to perfusion methods and will extend the use of PHH in research and drug development.

**Supplementary Information:**

The online version contains supplementary material available at 10.1007/s44164-025-00097-4.

## Introduction

Primary human hepatocytes (PHH) are considered the gold standard for the study of hepatocyte biology, including metabolism and toxicology [1–3]. However, the widespread use of PHH is constrained by limited access to human liver tissue, the necessity for large samples, and the high cost and specialised expertise required for perfusion isolation techniques. This has led researchers to use cancer-derived cell lines, such as HepG2, HepaRG and Huh7. However, these do not fully recapitulate the physiological and functional properties of PHH. For example, cell lines display altered glucose and fatty acid metabolism to support abnormal proliferation [4] and HepG2 cells have lower cytochrome P450 metabolism [5]. Translating laboratory findings into effective clinical treatments remains a major challenge in biomedical research, particularly in the field of liver disease. A key factor contributing to this gap is the lack of preclinical models that accurately replicate the complex physiology of the human liver in both healthy and diseased states. The limitations of using animal models and cell lines are illustrated by the drug-induced liver toxicity of fialuridine [6], nefazodone [7] and trovafloxacin [8], which only emerged during clinical trials. These failures underscore the urgent need for more physiologically relevant, human-based liver models that can better predict drug safety and efficacy. In this context, improving access to PHH, which retain many of the metabolic and functional characteristics of the native liver, is of critical importance to bridge the translational gap and enhance the reliability of preclinical research.

The current benchmark for PHH isolation is the enzymatic two-step perfusion protocol [9, 10]. This is based on the use of collagenase to disaggregate cells [11] and perfusion of the enzyme through the vasculature to ensure uniform distribution across the tissue [12]. Initially optimised for rat liver [9–12], the two-step perfusion protocol was later adapted for human liver specimens [13, 14]. The procedure begins with vascular perfusion of calcium (Ca²⁺)-free chelating buffer to disrupt intercellular junctions and remove red blood cells (RBCs), followed by perfusion with collagenase to degrade extracellular matrix proteins and release PHH. The reintroduction of Ca^2+^ in the second step is crucial for collagenase activation, increasing enzymatic activity and improving tissue digestion [9, 10].

Currently, whole livers unsuitable for orthotopic transplantation or resected liver tissue from cancer patients are used for PHH isolation using the two-step perfusion protocol. Reported yields from perfusion protocols average 7.56 × 10^6^ cells/g tissue, with a 70 ± 6% average viability (Table Supporting Information (S)1). Although effective, this method requires large liver samples (80–120 g), with accessible blood vessels and an intact Glisson’s capsule [15–18]. These requirements limit the availability of suitable samples, while the need for perfusion apparatus and large volumes of reagents further increases operational costs. These challenges prompted the development of an alternative non-perfusion isolation method that combines mechanical and enzymatic digestion. The protocol involves mechanical dicing, EGTA and collagenase digestion, followed by an RBC lysis step, resulting in yields of 0.64 ± 0.19 × 10^6^ cells/g tissue, with 73% viability [17]. The presence of hepatocytes was confirmed by expression of albumin, cytokeratin (CK)18 and CYP3A4/5/6 [17]. However, as with most hepatocyte isolation studies [16, 19–21], purity and the presence of other liver cell types were not quantified [17]. This non-perfusion isolation method remains the only published description to date, highlighting the need for further development. The Miltenyi Biotec human tumour dissociation kit [22] has also been used to isolate PHH (personal communication). While these efforts were reportedly successful (data unpublished), further experimental validation is required.

The review of published isolation studies summarised in Table S1, revealed that PHH purity is rarely reported. While hepatocyte identity is often validated by protein markers or the secretion of hepatocyte-associated proteins, the proportion of hepatocytes within the total cell population is seldom specified. CK8 and CK18 are commonly used to confirm hepatocyte identity [16, 17, 23, 24]. However, cholangiocytes, while typically expressing CK7 and CK19, can also express CK8 or CK18 [25, 26]. Therefore, CK8 and CK18 alone are insufficient to accurately assess hepatocyte purity. Only one study used the hepatocyte-specific marker albumin [27] to assess purity, reporting 94% purity. However, this was based on only 4 out of 648 liver isolations [18], limiting its generalisability. These findings emphasise the need for more robust and standardised approaches to evaluate hepatocyte purity in PHH isolation protocols.

In order to overcome these challenges and improve access to human hepatocytes for research, we sought to develop a cost-effective isolation protocol tailored for small liver samples (≤ 21 g) unsuitable for perfusion. We compared two non-perfusion methods [17, 22] with three modified protocols, informed by insights from perfusion [9, 16–18, 20, 21, 23, 28] and non-perfusion methods [17], to identify the most effective approach. We characterised the performance of these protocols in terms of hepatocyte yield, viability and cellular composition, and evaluated their potential for PHH isolation. Importantly, our novel isolation method also allows for the simultaneous generation of human precision-cut liver slices (hPCLS), maximising the utility of valuable human specimens.

## Methods

### Reagents and materials

The reagents, materials and buffer compositions used in this study are detailed in Tables S2 - S4.

## PHH isolation protocols

### Tissue samples

Human liver samples were obtained from patients who had undergone surgical liver resection and assessed histologically by a pathologist. This study was approved by the local Research Ethics Committee established by the Health Research Authority (REC reference 21/YH/0206 and REC reference 22/SC/0429) and conducted in accordance with the Declaration of Helsinki.

### Cell culture plate and buffer Preparation

Buffers and media were prepared as described in Table S4. Plates were coated with type I collagen according to Table S5 and incubated at 37 °C, 5% CO_2_ for a minimum of 1 h before use.

### Protocols

Protocol 1 = Published Green et al. non-perfusion PHH protocol [17].

Protocol 2 = Miltenyi Biotec, human tumour dissociation kit [22].

Protocol 3a = A hybrid protocol of the Miltenyi Biotec [22] and Green et al. [17]. protocol using diced liver tissue.

Protocol 3b = A hybrid protocol of the Miltenyi Biotec [22] and Green et al. [17]. protocol using vibratome-sliced liver tissue.

Protocol 4 (final) = Final optimised PHH isolation protocol.

**Fig. 1:**
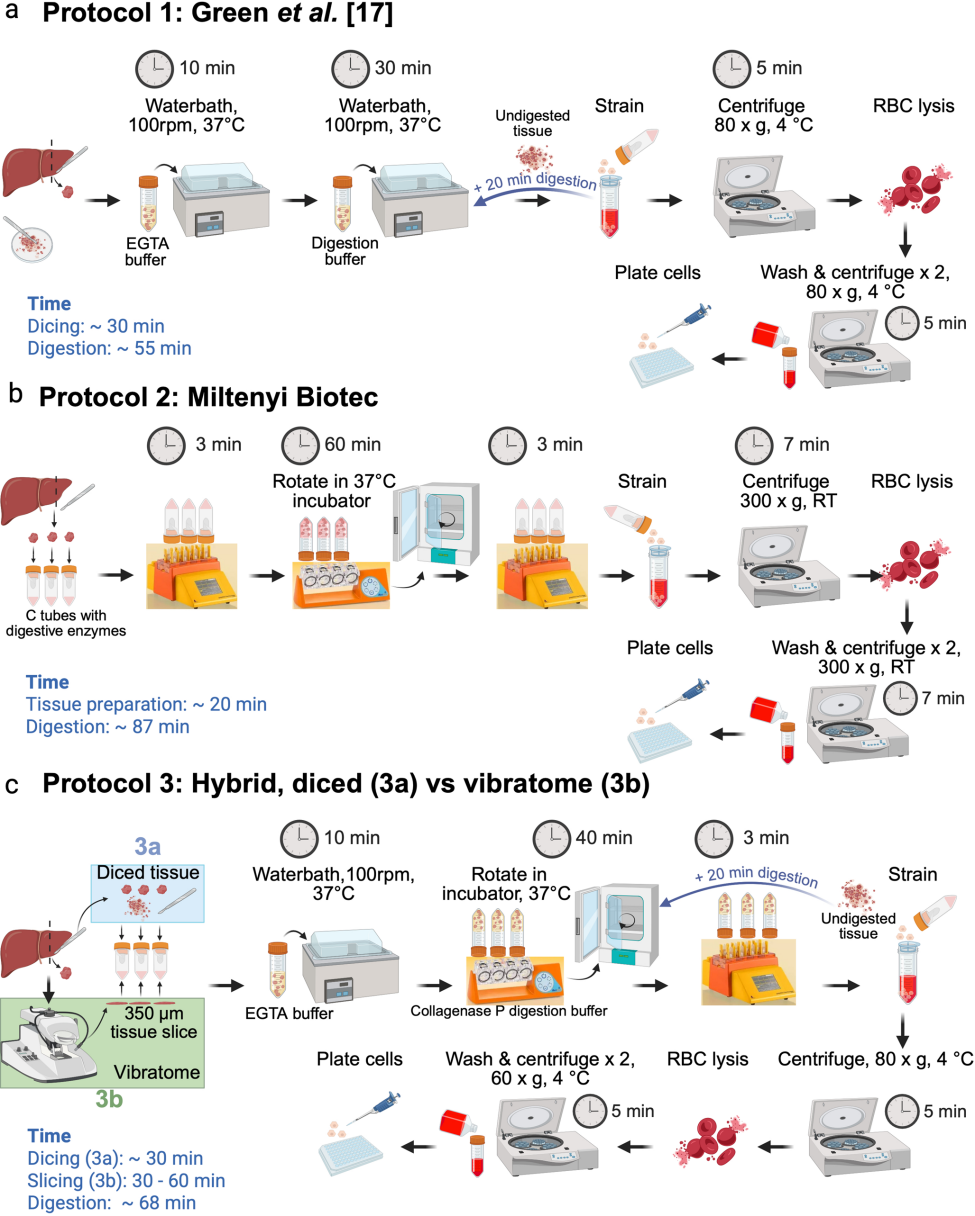
Protocols used in the development of our optimised PHH isolation protocol

Only Protocol 4 (final) is described in the methods section. Protocols 1–3 are illustrated schematically (Fig. 1a **− **1c) and in the section immediately below. When possible, comparisons for protocols 1, 2 and 3 were performed on the same liver sample.

### Protocols used in the development of our final optimised isolation protocol

Protocol 1, as described by Green et al. [17], was performed as shown in Fig. 1a. Protocol 2 was performed as described in Fig. 1b, following the human tumour dissociation kit manual [22] with the omission of the second run on the GentleMACs tissue dissociator. Protocol 3 (Fig. 1c) was a novel hybrid approach that combined elements from Protocol 1 (Green et al. [17]). and Protocol 2 (Miltenyi Biotec). In protocol 3, liver specimens were subjected to a two-step digestion (1. EGTA, 2. Collagenase) with a 40 min collagenase incubation, incorporating the MACsmix tube rotator and the GentleMACs dissociator. Additional modifications in protocol 3 included the addition of BSA to both EGTA and digestion buffers for cell protection [17, 23, 29] and collagenase P to enhance PHH viability [23, 29]. Protocol 3 was applied to diced and vibratome-sliced liver samples.

Schematic representation of **(a)** Protocol 1 (Green et al. [17]*)* **(b)** Protocol 2 (Miltenyi Biotec) and **(c)** Protocol 3 (hybrid intermediate), which compared the isolation of PHH from diced liver (3a, blue box) and vibratome cut liver slices (3b, green box). Tissue preparation, dicing, slicing and digestion time indicated in blue, but varied depending on tissue characteristics.

###  Tissue collection and preparation for protocol 4

**Fig. 2:**
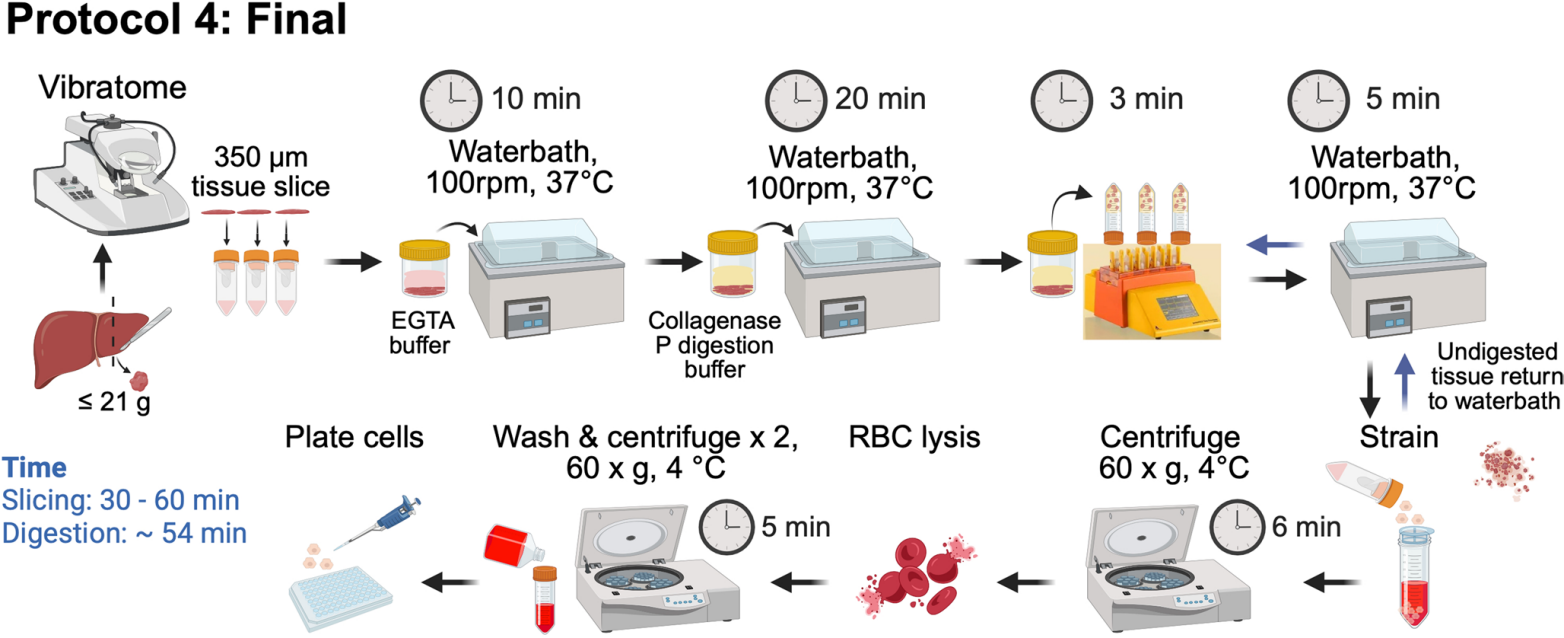
Schematic of Protocol 4, the final optimised non-perfusion PHH isolation protocol

**Fig. 3:**
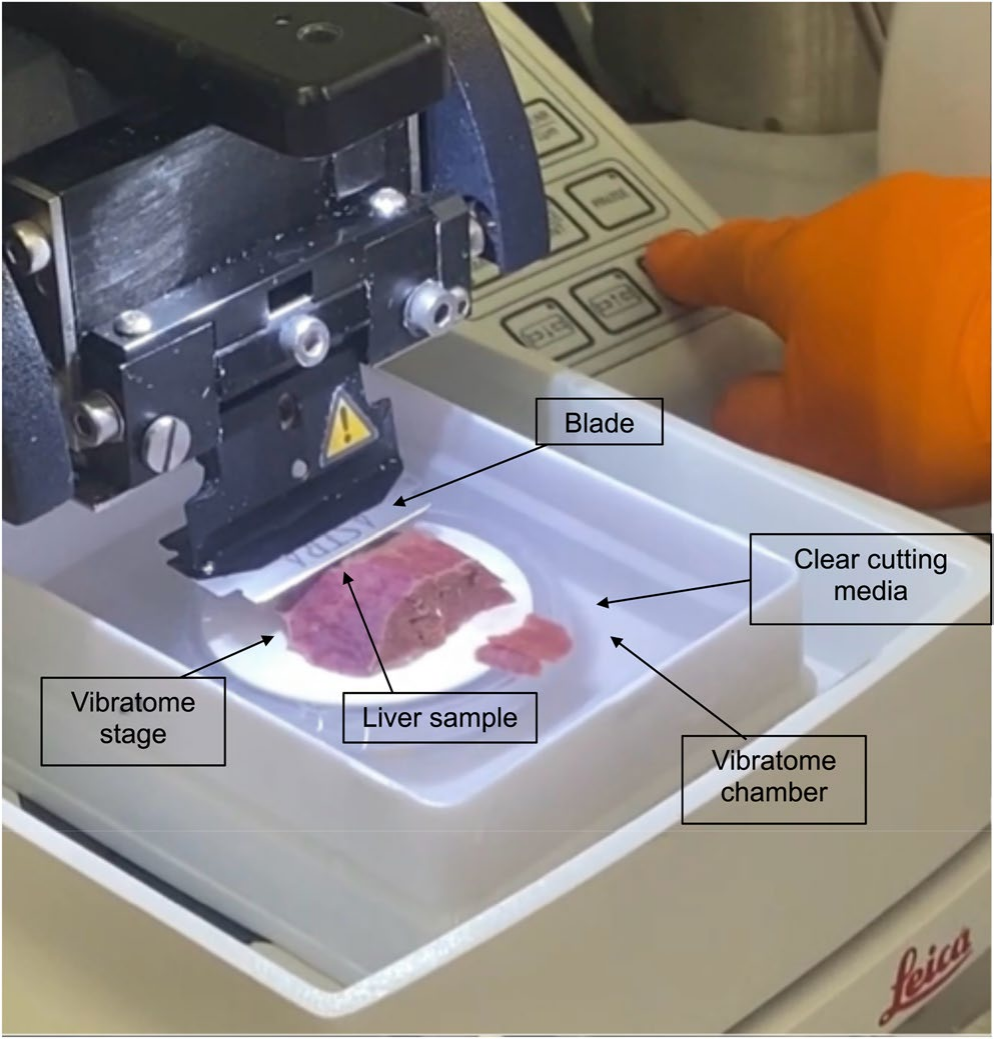
Image of a human liver tissue sample being cut on the vibratome

Liver samples macroscopically free of cancer were collected within 20 min after surgical resection and transported to the lab in MACS tissue storage solution on ice. Excess liquid was removed using tissue paper and the sample was superglued to the vibratome stage. After approximately 1 min of drying at room temperature (RT), the stage was placed on the vibratome, 4 °C vibratome cutting media was added to the vibratome chamber, a blade attached and 350 μm tissue slices cut (Fig. 2 and Fig. 3). Slices were collected with sterile forceps and transferred to storage media on ice. The tissue slices were transported on ice to a biological safety cabinet. After draining the storage media, the slices were placed in a 10 cm^2^ culture dish on ice and large blood vessels and fibrotic areas were excised using a scalpel. The sample was then washed with 4 °C HBSS and weighed.

### *Notes*

- The vibratome stage should be autoclaved after each use.
- The sample may need to be trimmed to match blade width.
- Allow sufficient time for the superglue to dry. Avoid introducing superglue into the storage media, as it is toxic.
- Change vibratome cutting media in the vibratome chamber if blood obscures the sample.

#### Protocol 4 tissue digestion

Tissue slices were transferred into a specimen container with pre-warmed 37 °C EGTA buffer, then placed in a 37 °C water bath with shaking (100 rpm) for 10 min. Simultaneously, the Miltenyi MACsmix C tubes (henceforth referred to as C tubes) were pre-warmed in the 37 °C water bath. The EGTA buffer was drained, and the tissue slices were washed once in RT HBSS. Pre-warmed 37 °C digestion buffer was added to the specimen container with the slices (45 mL for up to 10 g tissue). The specimen container was returned to the 37 °C water bath with shaking (100 rpm) for 20 min. The specimen container was removed from the water bath and 8 tissue slices (approximately 3.5 g of tissue) were transferred to each C tube with the same 37 °C digestion buffer (8–10 mL/tube). The C tubes were then transferred to a MACS Dissociator. The GentleMACs “h_tumour_2” program was run once. “h_tumour_2” (234 rounds per run (rpm), 36 s) is a pre-defined GentleMACs tissue dissociation protocol described by Miltenyi (located under “Miltenyi” folder on the GentleMACs Dissociator). After dissociation, the digested tissue was transferred from the C tubes to the specimen container and returned to the 37 °C water bath with shaking (100 rpm) for 5 min. A 100 μm strainer was placed on a 50 mL falcon tube and wet with RT WME. The specimen container was removed from the water bath and the top ¾ of the digested tissue suspension was poured through a strainer. 25 mL of cell suspension was collected in each 50 mL falcon tube, 25 mL of RT WME was added to the remainder of the falcon tube, and the tubes were placed on ice immediately. Fresh pre-warmed digestion buffer was added to the remaining ¼ of incompletely digested tissue suspension and returned to the 37 °C water bath with shaking (100 rpm) for 5–10 min. The specimen container was removed from the water bath, and the tissue suspension transferred to a C tube (10 mL/tube). The C tubes were transferred to the MACS Dissociator and the “h_tumour_2” program was run once more. The C tubes were returned to the 37 °C water bath and the remaining digested cell suspension of each C tube was strained one at a time. Again, 25 mL of cell suspension was collected per falcon tube, 25 mL of RT WME was added to the remainder of the falcon tube and the tubes were placed on ice. All subsequent buffers/media and cells were kept at 4 °C on ice until the final step when cells were resuspended in RT plating media. This minimises cellular temperature fluctuations.

#### *Notes*

- Ensure the specimen container is large enough for the slices to move freely and tissue is completely covered with buffer. The tissue digests better in larger containers in which it is free to move. Therefore, tissue slices are returned to the specimen container to digest when possible.
- Several falcon tubes and strainers will be required, depending on the size of the tissue.
- In order to prevent cell death and avoid over-digestion, the digested fraction (top **¾** of the specimen container) is strained, neutralised with WME and promptly placed on ice. Only the remaining **¼** of incompletely digested tissue undergoes further incubation with the digestion buffer.
- The volume of additional fresh digestion buffer required will depend on the amount of undigested tissue remaining. Usually, 5–15 mL is sufficient.
- If tissue is fully digested at the initial straining step, as indicated by a cloudy suspension and absence of visible tissue fragments, additional digestion time and GentleMACs Dissociator run are not required. The duration of any additional digestion depends on the tissue; it is recommended to re-assess the suspension after 5 min for cloudiness and tissue fragments.

### Protocol 4 cell processing and plating

All falcon tubes (henceforth referred to as tubes) were centrifuged (60 x *g* for 6 min, 4 °C). It is recommended to aspirate the supernatant with a 50 mL stripette to avoid the soft pellet also being discarded by pouring. RBC lysis buffer (1 mL) was gently added to each tube and the cell pellet was resuspended gently. The tubes were incubated on ice for 1 min and then 40 mL of 4 °C PBS was added to each tube to neutralise the lysis buffer. All tubes were centrifuged (60 x *g* for 6 min, 4 °C) and the pellet was washed with 45 mL of 4 °C WME twice. The cell suspension was passed through a 100 μm strainer. The filtered suspension was centrifuged at 60 x *g* for 6 min (wash 1) and 50 x *g* for 5 min (wash 2) at 4 °C, then resuspended in 3 mL RT plating media. Cells were counted to determine the seeding density. Cells were counted and viability assessed using Acridine Orange/Propidium Iodide staining according to the manufacturer’s instructions. Cells were counted using a LUNA-FL™ Dual Fluorescence Cell Counter. The viability and cell count were only recorded for cells ≥ 18 μm in size.

The collagen I-coated plates were washed twice with PBS and cells were seeded at a density of 250,000/cm^2,^ using the volumes shown in Table 1. Seeded plates were placed in a 37 °C incubator with 5% CO_2_. After 4 h the cells were checked for adherence and 80% of the total well volume was gently aspirated by manual pipetting. Fresh medium was added gently (the same volume that was removed) and the plate was returned to the 37 °C incubator with 5% CO_2_. After 18–24 h the plating media was fully aspirated, and the cells were washed with prewarmed 37 °C PBS + pen-strep twice and 37 °C culturing media (HCM) was added to the cells.

**Table 1:** Volumes of plating media and cell suspension to add

Plate format	Volume plating media to add to each well	Volume of cell suspension	Total volume in well
12 well	500 µL	500 µL	1 mL
24 well	400 µL	400 µL	800 µL
96 well	50 µL	50 µL	100 µL

#### *Notes*

- Carefully resuspend the pellet by holding the falcon tube in your hand and slowly adding media while rotating the tube until the pellet dissolves. Aggressive pipetting of cells will cause cell death.
- Careful resuspension is crucial at all steps.
- The vibratome slicing procedure was performed under non-sterile conditions. The addition of pen-strep in cutting and tissue slice storage solution, as well as antibiotic-antimycotic in plating media, reduced the risk of contamination.
- Ensure the collagen I-coated plates are fully prepared by the time the cells are suspended and counted in the plating media to maximise viability. Quick plating after counting is crucial for maintaining survival. Following at least 1 h incubation of collagen I-coated plates at 37 °C, 5% CO_2_, wash twice with PBS and add the volume specified in column 2 of Table 1 to each well during the digestion incubation or centrifugation. Avoid allowing the collagen I-coated plates to dry out at any point. After counting, gently plate cell suspension using volumes in column 3, Table 1.
- PHH adhere within the first 4 h, but complete adherence and flattening can require an additional 12–24 h. Only 80% of the media is removed after 4 h to avoid detachment of lightly adhered cells.
- If the liver is steatotic, it is advised to centrifuge at 80 x *g* for 6 min for all steps, only remove 50% of the total well volume after 4 h and wait 24 h before the initial change. Higher lipid content may increase the time required for cell adhesion [17].

### Cell culture maintenance

Cell culture media was replaced every 24 h. Media was pre-warmed to 37 °C before use. The culture plate was tilted, spent media was slowly aspirated from the edge of the well and fresh media was gently added. The plate was then returned to a 37 °C incubator with 5% CO_2_.

### Exclusion criteria for experiments

Liver samples < 2 g, or if more than 50% of the specimen was cauterized, or specimens with > 6 large bile ducts per 5 g tissue were excluded from this study. The high density of bile ducts in a small tissue area hindered the cutting of full vibratome slices.

### Immunofluorescent staining

Isolated cells were characterised by immunofluorescent staining of liver cell markers. All reagents used are listed in Tables S6 and S7. Isolated cells cultured in Ibidi imaging dishes were washed twice with PBS, then fixed in 4% formaldehyde for 20 min at RT. Cells were washed twice with PBS and permeabilised with 0.1% Triton X-100 for 5 min at RT. After 2 additional washes with PBS, cells were blocked with 1% BSA/10% normal goat serum in 0.1% Tween for 1 h at RT. Cells were then incubated with primary antibodies or respective isotype controls (Table S6) in antibody dilution buffer overnight at 4 °C. Cells were washed 3 times for 5 min in PBS at RT. If required, cells were then incubated with secondary antibodies in antibody dilution buffer for 1 h at RT, followed by an additional 3 PBS washes of 5 min at RT. If used, LipidSpot was diluted 1:1000 in PBS, cells were then incubated for 30 min at RT. Cell nuclei were stained with DAPI for 12 min at RT. Finally, cells were washed briefly in PBS and 3 drops of mounting media were added to each well.

### Image acquisition and analysis

CZI z-stack images were acquired using a Zeiss LSM 980 confocal microscope with a Plan-Apochromat 10X air objective with Numerical Aperture 0.45, 20X air objective with Numerical Aperture 0.8 or 63X with Numerical Aperture 1.4 oil DIC M27 objective. Maximum projection images were generated in ImageJ (version:2.3.0/1.53p. *RRID*: SCR_003070) and imported into QuPath (*RRID*: SCR_018257). Positive cell selection on DAPI-stained cells was used to calculate the total number of cells in each image. Each marker was thresholded for positive staining and the percentage of positive cells was calculated using the total DAPI cell count. ImageJ was used to scale the bar and prepare images for publication.

### Albumin ELISA

Cryopreserved PHH (Lonza, #HUCPG) were plated according to the manufacturer’s instructions. Culture supernatants from isolated and commercial PHH were collected after 24 h and stored at − 80 °C. Albumin concentrations were quantified using a Human Albumin ELISA Kit (Thermo Fisher Scientific, EHALB) following the manufacturer’s instructions. Absorbance was measured using a FLUOstar Omega plate reader.

### Periodic acid schiff (PAS) staining

All steps were performed at RT. Cells were fixed in 350 µL of 4% formaldehyde for 12 min, washed twice with PBS and stained using a PAS staining kit (ab150680). Cells were incubated with Periodic Acid solution for 5 min, rinsed in distilled water for 2 min, and stained with Schiff’s reagent for 12 min. After an additional 1 min rinse in distilled water, cells were counterstained with hematoxylin for 3 min and washed 3 times for 5 min each in distilled water. Imaging was performed using Leica DM IL LED/Fl Microsystems and processed using LAS X software. PAS-positive cells were identified by dark magenta cytoplasmic staining.

### Graphical schematic and statistics

All graphical schematics were created using Biorender.com. Statistical analysis and graphs were performed using GraphPad Prism version 10.2.1. The family-wise alpha threshold was 0.05 and a 95% confidence level. The specific statistical tests used are described in the corresponding figure legends. Significance levels are indicated as *p < 0.05, **p < 0.01, ***p < 0.001, ****p < 0.0001. All data are shown as mean ± SEM.

## Results

### Patient characteristics

PHH were isolated from 13 liver resections. The cohort included 10 males (M), 2 females (F), and 1 individual whose gender data were unavailable. The mean age was 64 years (range 44–80 years) and the mean BMI was 29.2 kg/m^2^ (range 23–41 kg/m^2^). Comorbidities included type 2 diabetes (*n* = 2), ischaemic heart disease (*n* = 1) and atrial fibrillation (*n* = 1). The indications for liver resection were metastatic colorectal adenocarcinoma (*n* = 8), cholangiocarcinoma (*n* = 3) and neuroendocrine liver metastasis (*n* = 2). Nine patients exhibited liver steatosis, while 2 had fibrosis and 2 presented with normal liver histology. 11 patients had undergone preoperative chemotherapy. A summary of these data is shown in Table 2.

**Table 2:** Details of patient characteristics for each isolation protocol

Protocol	Protocol 1 Green	Protocol 2 Miltenyi Biotec	Protocol 3a Hybrid: diced	Protocol 3b Hybrid: vibratome	Protocol 4 Final
n	2	2	2	2	9
Age	56 ± 2.0	49 ± 5.0	74 ± 6.5	74 ± 6.5	69 ± 4.0
BMI	30 ± 2.2	30 ± 2.1	31 ± 2.0	31 ± 2.0	29 ± 1.9
Liver histology, n (% of total)					
Normal		1 (50%)			2 (22%)
Steatotic	1 (50%)	1 (50%)	1 (50%)	1 (50%)	6 (67%)
Fibrotic	1 (50%)		1 (50%)	1 (50%)	1 (11%)
Chemotherapy, n (% of total)	1 (50%)	1 (50%)	1 (50%)	1 (50%)	7 (77%)
Weight of tissue (grams)	10 ± 1.8	10 ± 2.0	3.7 ± 0.5	2 ± 0.3	8.5 ± 2.0

### Vibratome slicing and reduced digestion time enhance hepatocyte yield

The efficiency of protocols 1, 2, 3a, 3b and 4 was evaluated by immunostaining to characterise the isolated cell populations. Hepatocytes were identified using albumin and CK8, cholangiocytes by CK8 and CK19, and mesenchymal cells by vimentin [30]. To assess the proportion of CK8-positive cells that were not hepatocytes, the percentage of double-positive CK8 and CK19 cells (CK8/CK19) was quantified. As albumin was the only hepatocyte-specific marker, it was used to assess purity.

**Fig. 4:**
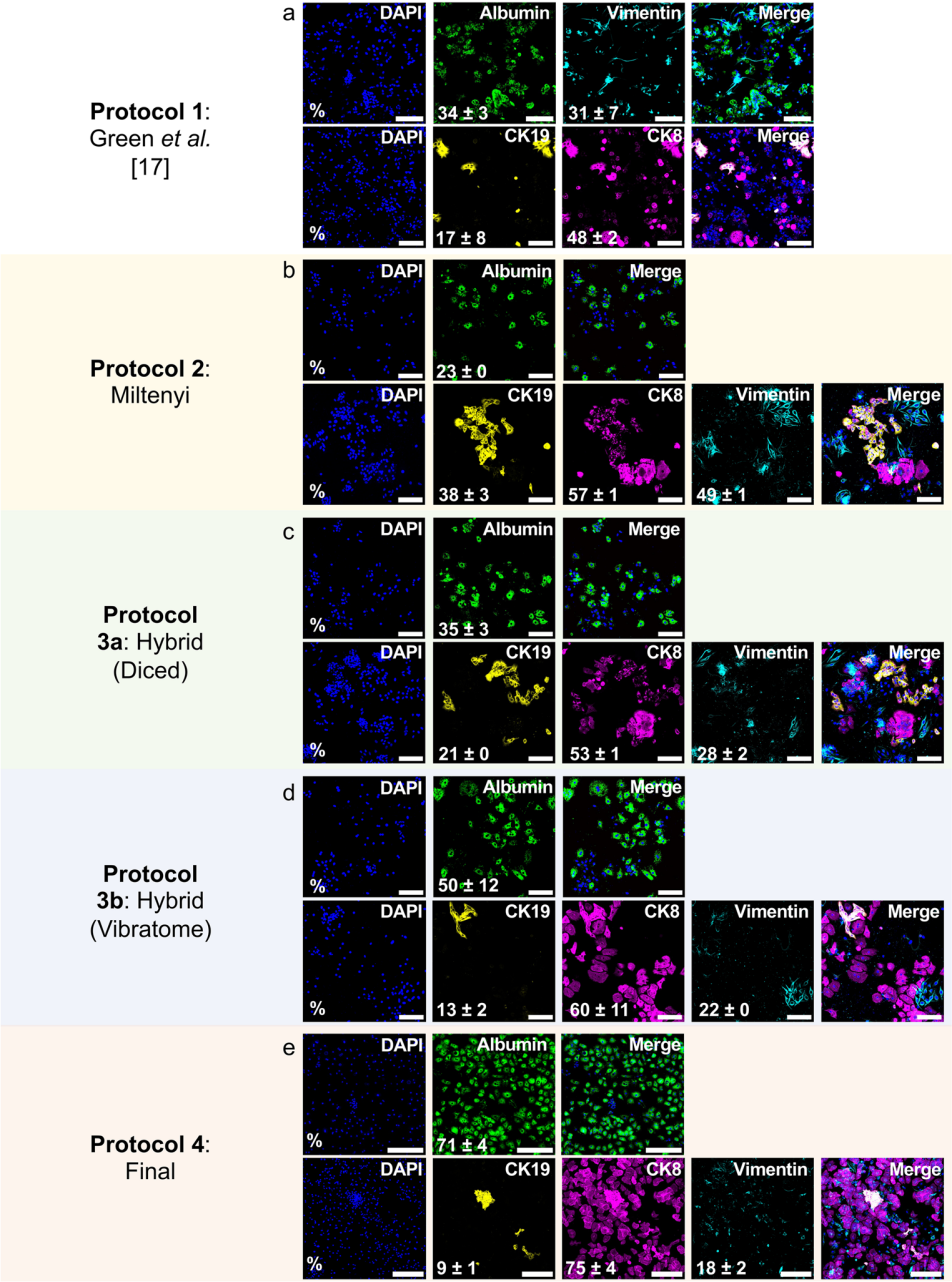
Optimised non-perfusion protocol 4 (final) improves the yield of albumin-positive cells. Representative immunofluorescent images of cell populations isolated using (**a**) Protocol 1 (Green et al. [17], *n* = 2), (**b**) Protocol 2 (Miltenyi Biotec, *n* = 2), (**c**) Protocol 3a (Hybrid: diced, *n* = 2), (d) protocol 3b (Hybrid: vibratome, *n* = 2) and (**e**) protocol 4 (final, *n* = 9). Hepatocytes were identified by albumin (Green) and CK8 (magenta), cholangiocytes by CK8 (magenta) and CK19 (yellow), and mesenchymal cells by vimentin (cyan). Percentages of marker-positive cells are displayed in white in the bottom left corner of each image. Scale bar = 100 μm. CK19 = cytokeratin 19. CK8 = cytokeratin 8

Protocol 1 (Green et al. [17])., resulted in 34 ± 3% albumin-positive, 48 ± 2% CK8-positive, 17 ± 8% CK19-positive, and 31 ± 7% vimentin-positive cells (Fig. 4a). In comparison, protocol 2 (Miltenyi Biotec) isolated a cell population that was 23 ± 0% albumin-positive, 57 ± 1% CK8-positive, 38 ± 3% CK19-positive, and 49 ± 1% vimentin-positive (Fig. 4b). Neither protocol yielded more than 35% albumin-positive cells, prompting the development of a hybrid protocol (protocol 3).

Protocols 1 and 2 involved manual tissue dicing before digestion. Therefore, to assess the impact of tissue processing, the hybrid protocol was applied to both diced liver (protocol 3a) and vibratome cut-liver slices (protocol 3b). Protocol 3a only yielded 35 ± 3% albumin-positive cells, whereas protocol 3b achieved 50 ± 12% albumin-positive cells. Immunofluorescent quantification of all cell populations is presented in Table 3.

**Table 3:** Summary of cell population isolated using each protocol as measured by immunofluorescent staining

Protocol	Protocol 1 Green	Protocol 2 Miltenyi Biotec	Protocol 3a Hybrid: Diced	Protocol 3b Hybrid: Vibratome	Protocol 4 Final
Purity (Albumin %)	34 ± 3	23 ± 0	35 ± 3	50 ± 12	71 ± 4
CK8%	48 ± 2	57 ± 1	53 ± 1	60 ± 11	75 ± 4
CK19%	17 ± 8	38 ± 3	21 ± 0	13 ± 2	9 ± 1
Vimentin %	31 ± 7	49 ± 1	28 ± 2	22 ± 0	18 ± 2
CK8/CK19%	17 ± 8	38 ± 3	21 ± 1	14 ± 2	9 ± 1

Extended digestion times can increase non-hepatocyte yields and cause proteolytic damage to PHH, compromising viability [23, 29]. Therefore, tissue digestion time was shortened from 40 to 25–30 min, based on completion of tissue dissociation at 25 min (Fig. 2). Additionally, instead of using C tubes on the MACSmix rotator, tissue slices were incubated in a specimen container in a shaking water bath, which maintained the optimal temperature for collagenase activity [17, 18, 29]. After these changes, protocol 4 (final) yielded a population of cells that was 71 ± 4% albumin-positive, 75 ± 4% CK8-positive, 9 ± 1% CK19-positive, and 18 ± 2% vimentin-positive (Fig. 4e; Table 3).

#### Cells isolated using protocol 4 (final) exhibit key characteristics of PHH

**Fig. 5:**
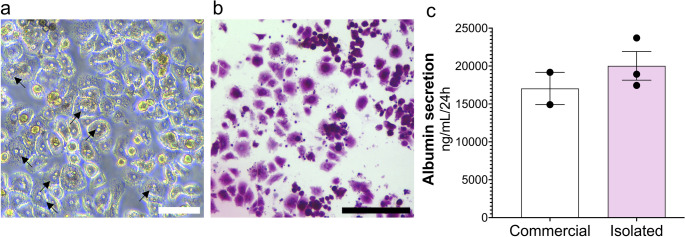
PHH isolated using protocol 4 (final) display characteristic morphology and glycogen storage (**a**) Brightfield image of isolated PHH, showing polygonal morphology and multinucleation (black arrows) (**b**) PAS staining in isolated cells (*n* = 3). Scale bar = 100 μm

Brightfield imaging revealed typical PHH polygonal morphology and multinucleation in the isolated cells (Fig. 5a). The isolated PHH exhibited strong PAS positivity (Fig. 5b), confirming their capacity for glycogen storage, a key metabolic function of hepatocytes [31, 32]. Additionally, albumin levels were quantified in 24 h supernatants. Secretion by PHH isolated using protocol 4 (final) were comparable to those of commercially sourced PHH (Fig. 5c). Together, these findings demonstrate that the isolated PHH retained key morphological features and metabolic functions characteristic of human hepatocytes, supporting their suitability for downstream experimental applications. Additionally, this method was used to generate vibratome-cut hPCLS that can be cultured and maintained ex vivo for at least five days (Fig. S1).

#### Protocol 4 significantly enhances hepatocyte yield and purity

**Fig. 6:**
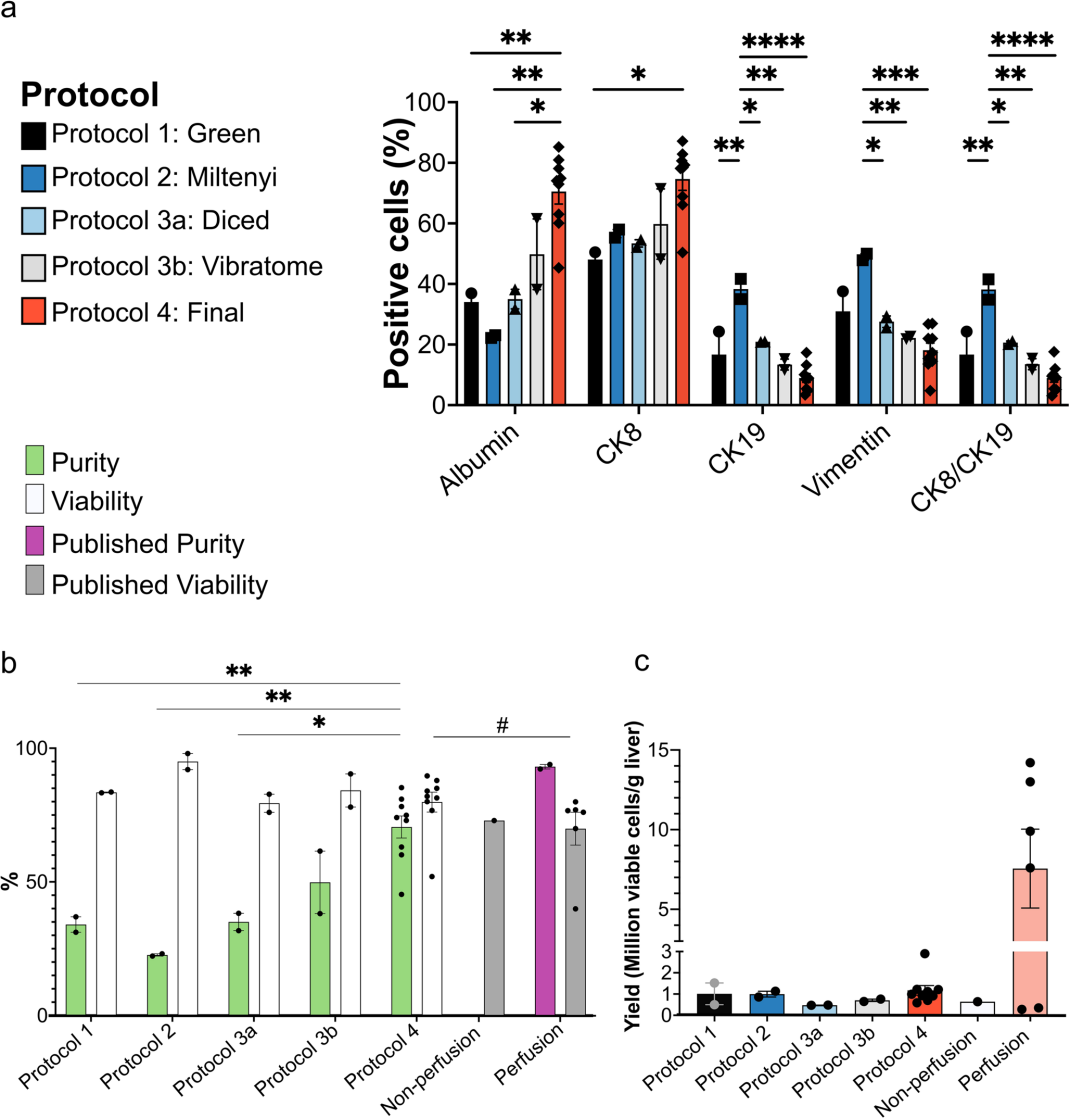
Protocol 4 (final) improves the yield of viable PHH compared to previous non-perfusion protocols

Next, we evaluated the impact of protocol optimisation on hepatocyte yield, purity and viability (Fig. 6). Across all protocols, the percentage of CK8/CK19 double-positive cells closely matched that of CK19-positive cells alone (Fig. 6a; Table 3), confirming that CK8 was not specific to hepatocytes, whereas CK19 serves as a specific marker for cholangiocytes. These findings reinforce our decision to use albumin as a more accurate marker for assessing hepatocyte purity.

Protocol 2 (Miltenyi Biotec) achieved the highest overall cell viability (Fig. 6b; Table 4) but yielded the fewest hepatocytes and the most mesenchymal cells (Fig. 6a; Table 3). Compared with protocol 2, protocol 3a resulted in a 17% reduction in cholangiocytes (*p = 0.0279), whereas protocol 3b achieved a 25% reduction in cholangiocytes (**p = 0.0021) and a 27% decrease in mesenchymal cells (**p = 0.0099) (Fig. 6a; Table 3).

**Table 4:** Summary of cell yields and viabilities isolated using each protocol

Protocol	Protocol 1 Green	Protocol 2 Miltenyi Biotec	Protocol 3a Hybrid: Diced	Protocol 3b Hybrid: Vibratome	Protocol 4 Final
Cell yield (cells/g tissue)	1.0 ± 0.5 × 10^6^	1.0 ± 0.1 × 10^6^	0.5 ± 0 × 10^6^	0.7 ± 0.1 × 10^6^	1.17 ± 0.2 × 10^6^
Viability (%)	83 ± 0	95 ± 3	79 ± 3	84 ± 6	80 ± 4

Further optimisations introduced in protocol 4 (final) significantly improved the isolation of hepatocytes by 37% compared to protocol 1 (**p = 0.0099), 48% compared to protocol 2 (**p = 0.0012), 36% compared to protocol 3a (*p = 0.0119), and 21% compared to protocol 3b. Protocol 4 also reduced cholangiocytes by 29% (****p < 0.0001), and mesenchymal cells by up to 31% compared to protocol 2 (***p = 0.0004, Fig. 6a; Table 3). Protocols 1 and 4 were applied to the same fibrotic liver specimen to directly compare protocol performance independent of inter-sample variability. Protocol 4 yielded more hepatocytes (Fig. S2), suggesting improved efficacy under challenging conditions.

Our final protocol achieved a PHH yield of 1.17 × 10^6^/g tissue, with an 80% average viability (Fig. 6b and c and Table 4), surpassing the previously reported non-perfusion method (0.64 × 10^6^/g tissue, 73% viability, no purity data) [17]. Although the yield is below the overall average for perfusion-based methods (7.56 × 10^6^ cells/g, Table S1), it outperforms several individual protocols included in that average [16, 20] (Fig. 6c). Our protocol achieved higher viability than the average (70%, *p = 0.0192) of published perfusion PHH isolation methods (Fig. 6b**).** As shown in Table S1, perfusion studies inadequately assessed purity, either not evaluating purity, using non-specific hepatocyte markers such as CK18 (92.3% purity [23, 29]), or assessing albumin expression in very few samples (4 of 648 liver specimens, 94% purity [18]), precluding reliable comparisons. Collectively, these results demonstrate that protocol 4 (final) represents a significant advancement in non-perfusion hepatocyte isolation methods.

**(a)** Quantification of albumin-, CK8-, CK19-, vimentin-, and double CK8/CK19-positive cells isolated using optimisation protocols 1–3b and the final optimised protocol 4 in this study (*n* = 13). **(b)** Mean cell viability and purity (% albumin-positive cells from (a)) from isolation protocols in this study (*n* = 13), compared with published protocols in Table S1 (perfusion *n* = 6, non-perfusion *n* = 1). No purity data were available for the published non-perfusion method. Asterisks (*) identify statistically significant differences in purity, and number sign (#) identifies a statistically significant difference in viability. **(c)** Mean cell yields from protocols 1–4 (*n* = 13), and published non-perfusion (*n* = 1) and perfusion methods (*n* = 6). Statistical significance was determined using a one-way ANOVA with Tukey’s multiple comparisons test for protocol 1–4 comparisons in (a) quantification of different cell populations, (b) viability and (c) yield, and by unpaired t-test for comparisons of protocol 4 with published perfusion methods in (b - c). Purity comparisons in (b) for protocols 1–4 were derived from one-way ANOVA analysis of albumin across protocols shown in (a). *p < 0.05.**p < 0.01. ***p < 0.001. ****p < 0.0001. CK19 = cytokeratin 19. CK8 = cytokeratin 8.

#### Highly viable cells can be isolated from steatotic liver samples

**Fig. 7:**
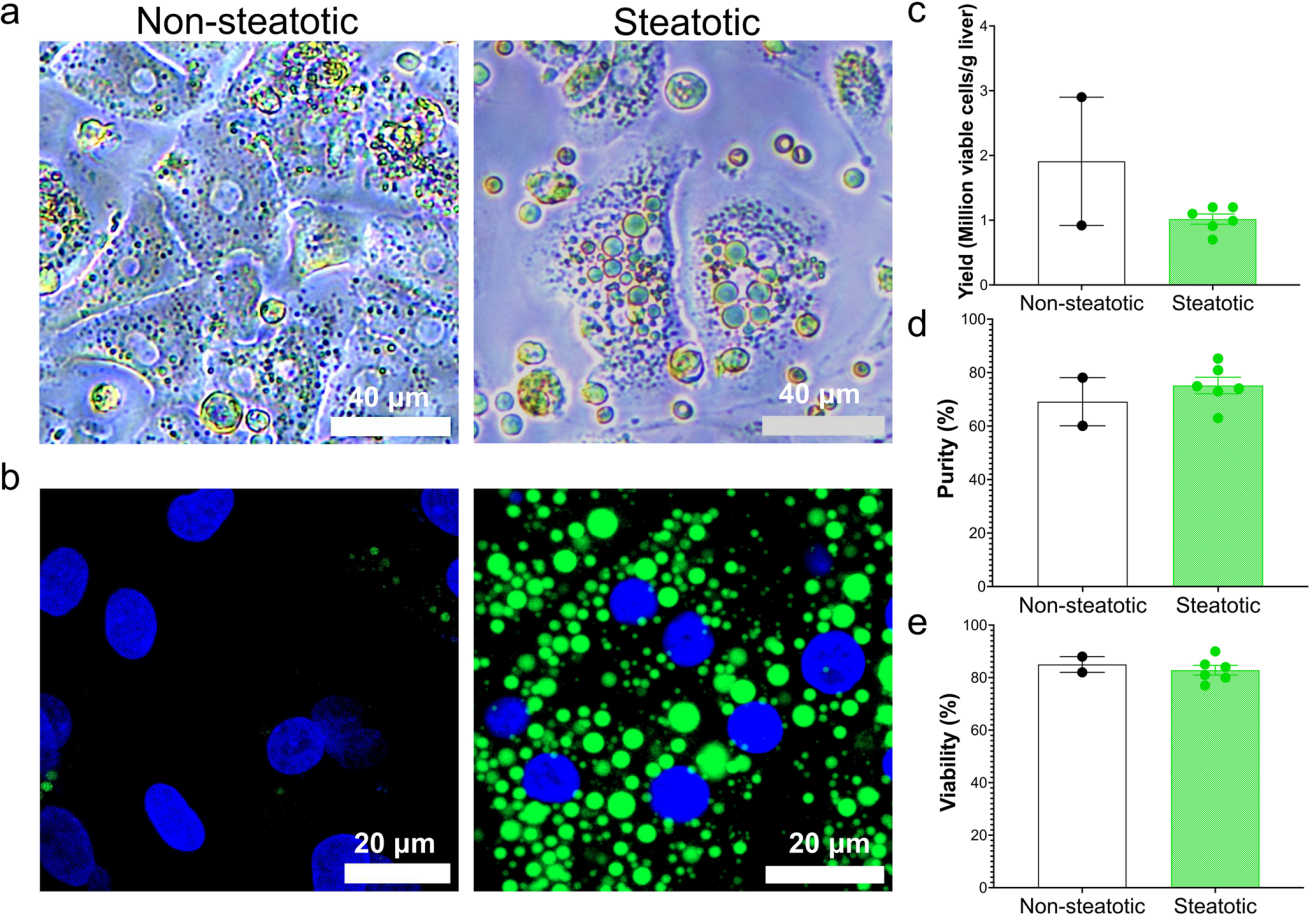
Steatosis reduces hepatocyte yield without compromising viability and purity. (**a**) Brightfield and (**b**) LipidSpot-stained immunofluorescent images of hepatocytes isolated from non-steatotic and steatotic liver samples. Cell yields (**c**), purities (**d**) and viabilities (**e**) from steatotic (*n* = 7) and non-steatotic (*n* = 2) liver specimens

The cells from patients with hepatic steatosis were characterised by the accumulation of lipid droplets (Fig. 7a, b). A trend towards reduced cell yield was observed in the steatotic samples, with the non-steatotic samples yielding 1.9 × 10⁶ cells/g compared to 1.0 ± 0.1 × 10⁶ cells/g in steatotic samples (Fig. 7c). However, purity (Fig. 7d) and viability (Fig. 7e) were similar between steatotic (purity: 75 ± 3%, viability: 83 ± 2%) and non-steatotic samples (purity: 69 ± 9%, viability: 85 ± 3%). The effect of steatosis on the viability and yield across other protocols in this study was also assessed (Fig. S3).

Most liver specimens were obtained from patients who had undergone neoadjuvant chemotherapy, however, no trend related to chemotherapy treatment was observed. Cell yield, purity and viability were comparable between chemotherapy-exposed specimens (yield: 1.3 ± 0.3 × 10^6^ cells/g, purity: 74 ± 4%, viability: 84 ± 2%) and the chemotherapy-naïve specimen (1.2 × 10^6^ cells/g, purity: 74%, viability: 81%, Fig. S4).

## Discussion

PHH represent the most physiologically relevant model for liver research. However, their limited availability precludes widespread use. While perfusion techniques provide high yields, suitable samples are extremely limited, and the associated costs can be prohibitive. Smaller resection specimens are more readily available but are not widely used due to the poor yield from existing isolation protocols. Therefore, we developed a novel protocol that does not require perfusion and leads to significantly higher yields of viable PHH.

We began by evaluating existing non-perfusion-based PHH isolation protocols: Gree*n et al.* [17] (protocol 1) and Miltenyi Biotec [22] (protocol 2). Protocol 2 was less efficient, likely due to the use of unspecified dissociating reagents and prolonged digestion times. We addressed this by replacing these elements with the shorter two-step digestion technique from protocol 1, resulting in a hybrid protocol (protocol 3). However, this did not achieve PHH purity above 50% and we further refined it to yield protocol 4 (final).

A key advance was the introduction of vibratome slicing, which minimised shear stress and increased tissue surface exposure to enzymatic digestion. Unlike earlier mechanical hepatocyte dissociation techniques, such as shaking liver slices with glass beads [33], which were abandoned due to mechanical stress-associated detrimental effects [18], vibratome slicing combined with the GentleMACS Dissociator improved PHH purity and yield without compromising viability. The isolated hepatocytes demonstrated preserved function and cellular integrity, as evidenced by carbohydrate metabolism, albumin synthesis and intact cell membranes, validating the effectiveness of protocol 4 (final). We attribute these improvements to the lateral saw-like motion of the vibratome, which likely reduces mechanical damage compared to dicing with a scalpel [34, 35], an important advantage given the sensitivity of hepatocytes to shear stress [29, 36]. Manual tissue slicing has been used for hepatocyte isolation from rat liver [28]. However, this approach produced thicker slices (0.5–1 mm), required longer digestion and the isolated cells were not characterised [28]. To our knowledge, this is the first application of vibratome-slicing for isolating human hepatocytes.

The next critical modification addressed inefficient digestion, by optimising digestion times and temperatures, resulting in a two-fold increase in hepatocyte yield compared to the other protocols. This improvement is likely due to more rapidly reaching and maintaining collagenase P at its optimal temperature of 37 °C [18, 29]. This enabled complete tissue dissociation in less time, reducing proteolytic damage to PHH associated with prolonged digestion [23, 29].

Tissue specimen weight has been shown to significantly impact successful hepatocyte isolation, with lower viabilities reported for small liver samples (< 50 g) [16, 17]. In contrast, our protocol consistently achieved high viability from liver samples averaging 8.5 ± 2.0 g. Although our yield did not match the average of perfusion-based studies, the latter required larger, encapsulated tissue samples with accessible vessels. In contrast, our protocol reliably isolated viable hepatocytes, unconstrained by sample size, the presence of vessels suitable for cannulation and an intact Glisson capsule to prevent leakage of fluids. This makes it particularly valuable for researchers with limited access to tissue samples. Additionally, this approach allows for the concurrent generation of hPCLS from the same tissue, maximising the utility of human samples.

The impact of steatosis on PHH viability and yield remains to be determined. While some authors reported a negative correlation between liver fat and hepatocyte yield and viability [37], others observed no impact of steatosis on viability, though yield was not assessed [17]. We observed a trend toward reduced hepatocyte yield [37] from steatotic compared to non-steatotic samples. However, viability and hepatocyte purity were comparable between groups, in agreement with Green et al. [17]. Importantly, the reduced viability and yield described previously were based on large, perfused specimens [37], whereas our protocol isolated hepatocytes with higher viability from smaller steatotic samples. However, these findings are based on limited numbers of non-steatotic samples. Green et al. [17] also employed a non-perfusion approach, suggesting these methods may better preserve the viability of hepatocytes isolated from fatty livers than perfusion techniques.

The lower yield from steatotic samples may result from high intracellular triglyceride content, which hinders cell pelleting during centrifugation at standard speeds (50–60 x *g*) and impairs cell adherence [16, 17]. This was addressed using strategies such as increased centrifugation speed (70–100 x *g*) and extended incubation periods before the initial media change [17]. PHH isolated from steatotic livers retain their in vivo steatosis phenotype, providing an invaluable resource for studying disease mechanisms. This circumvents the limitations of inducing damage in PHH from non-steatotic donors, which does not fully replicate native disease.

Our findings reinforce that CK8 alone is insufficient for assessing hepatocyte purity. As it is expressed by both hepatocytes and cholangiocytes, CK8 lacks the specificity required for accurate cell-type discrimination. Therefore, previous studies that have solely relied on the expression of CKs may have overestimated hepatocyte yield [23, 29]. In contrast, albumin emerged as the most specific and reliable hepatocyte marker in our study. We recommend that future studies adopt standardised criteria for purity assessment, with albumin-positivity as a minimum requirement to identify hepatocytes.

Our review identified published perfusion protocols report a mean hepatocyte purity of 93%. However, this was based on only two studies, one using a non-specific hepatocyte marker and the other assessing purity in only 0.6% of samples. Although our final protocol did not yield a completely enriched hepatocyte population, a purity of around 70% was achieved, consistent with the physiological proportion of hepatocytes in the human liver [38]. Attempts to improve purity using Percoll were unsuccessful (data not shown), aligning with previous studies demonstrating that Percoll is only beneficial for enhancing purity when initial viability is low and cell yields are high [16, 39]. While mixed cell populations may be seen as a limitation, they provide a more physiologically relevant environment for downstream applications. Additionally, co-culture systems have been shown to better preserve hepatocyte phenotype and function in vitro [40, 41]. Given the > 40% increase in purity compared to our starting protocols and the absence of published purity data for non-perfusion protocols, we believe that the 70% purity achieved from our non-perfusion protocol represents a significant advance.

Mature primary hepatocytes exhibit limited or no proliferative capacity under standard 2-dimensional (D) culture conditions [42], a recognised limitation. Strategies to address this include 3D culture, extracellular matrix scaffolds, soluble growth factors [43–45] and co-culture systems [46]. As our isolated cells comprised mixed hepatic cell types, paracrine interactions may have supported hepatocyte proliferation. Additionally, the PHH culture medium contained hydrocortisone and EGF, which have been shown to promote PHH expansion in vitro [47]. High viability was confirmed following isolation by Acridine Orange/Propidium Iodide staining, which distinguishes live from dead cells but does not assess proliferation. No clear evidence of cell-cycle re-entry was observed; however, this could be evaluated in future studies using proliferation markers such as EdU or Ki-67.

Despite the promising results of our optimised protocol, several limitations should be acknowledged. We only tested the published non-perfusion (protocol 1, Green et al..) [17] in samples from 2 donors. Nonetheless, our final protocol exceeded the yield and viability reported in the original publication. Our protocol also yielded more hepatocytes from a fibrotic sample compared to when we used protocol 1 (Green et al.). Only one of the liver samples processed using protocol 4 was obtained from a patient who had not undergone adjuvant chemotherapy, which may have introduced bias. Chemotherapy has been associated with enhanced hepatocyte yields [37], possibly by reducing extracellular matrix proteins (e.g. collagen), thereby facilitating more efficient enzymatic digestion [37]. However, using our final protocol, the yield from the chemotherapy-naïve specimen was comparable to the yield from chemotherapy-exposed specimens, suggesting no apparent bias. Furthermore, this is unlikely to limit reproducibility, as small surgical resections, especially those available for research, are commonly obtained from cancer patients who have undergone chemotherapy. Additionally, as we had immediate access to liver tissue following resection, the efficiency of the protocol using transported samples remains unknown. However, prolonged ischaemic times are known to reduce viable hepatocyte yield regardless of the isolation method [29]. Lastly, while our protocol provides an alternative to perfusion-based methods, it still requires some specialised equipment, such as a vibratome and a GentleMACS Dissociator. Whilst this equipment can be costly, they are more affordable than perfusion systems and these devices can be used more widely for cell isolation and tissue preparation for culture or histology. Importantly, vibratome-cut slices can also be cultured and maintained ex vivo for at least 5 days, supporting further applications in cell-cell interaction studies, drug toxicity screening and drug efficacy assessments.

In conclusion, we describe a refined protocol for isolating viable PHH from small liver samples, expanding the range of specimens suitable for hepatocyte isolation. Future work will focus on isolating and purifying non-PHH cell populations, as demonstrated in previous perfusion studies [23, 29]. This will enable further investigations into the roles of other cell types in both physiological and pathological conditions. Additionally, validating this protocol on larger liver samples (> 21 g) not suitable for perfusion will be important for further extending the samples suitable for PHH isolation.

## Supplementary Information

Below is the link to the electronic supplementary material.

Supplementary Material 1 (DOCX 5.55 MB)

## Data Availability

No datasets were generated or analysed during the current study.
