# Supplementary material for: High-yield isolation of primary human hepatocytes from small liver samples
Source: In Vitro Model. 2025 Nov 13;4(3-4):245–59. doi: 10.1007/s44164-025-00097-4 (PMC12909712; doi:10.1007/s44164-025-00097-4)
Supplement: Supplementary file 1 — Supplementary Material 1 (DOCX 5.55 MB) [file 44164_2025_97_MOESM1_ESM.docx]

Supplementary Information

High-yield isolation of primary human hepatocytes from small liver samples

**Author information**

Thea Guy^1^, Jia-Ling Ruan^2^, Carl Lee^1^, Kaitlyn Purdie^1^, David Johnson^4^, Alex Gordon-Weeks^3^, Jagdeep Nanchahal^1^

**Author affiliations**

**^1^The Kennedy Institute of Rheumatology, Nuffield Department of Orthopaedics, Rheumatoid and Musculoskeletal Sciences, University of Oxford, United Kingdom**

**^2^Department of Oncology, University of Oxford, United Kingdom**

**^3^The Nuffield Department of Surgical Sciences, University of Oxford, United Kingdom**

**^4^Department of Engineering Science, University of Oxford, United Kingdom**

Corresponding author: Jagdeep Nanchahal. Contact: [jagdeep.nanchahal@kennedy.ox.ac.uk](mailto:jagdeep.nanchahal@kennedy.ox.ac.uk)

**Submitted to In vitro models**

**Supplementary** **Table 1.** Table of PHH isolation papers published after 2011.

| **n** | **Protocol** | **Number of livers & mean liver tissue weight** | **PHH validation & purity** | **Viable PHH yield (cells/g tissue & viability** |
| --- | --- | --- | --- | --- |
| **Non-Perfusion** |  |  |  |  |
| Green *et al.* (2017)[1] | Combined mechanical & enzymatic digestion | **Number of livers:** 44  **Mean weight:** not specified. Samples ranged from 7.8 – 600 g | Western blotting: albumin, CK18, AFP, CYP3A4/5/7.  Urea secretion  IF: TMRM for viability  Purity: not specified | **Mean yield:** 0.64 ± 0.19 x 10^6^  **Mean viability:** 73 ± 13% |
| **Perfusion** |  |  |  |  |
| Pfeiffer *et al.* (2015),  Kegel *et al* (2016)[2, 3] | Two-step collagenase perfusion. | **Number of livers**: 12  **Weight:** not specified | IF: CK18 staining  Purity (CK18): 92.3 ± 3.2 % | **Mean yield:** 14.2 ± 6.6 x 10^6^  **Mean viability:** 76.6 ± 4.2 % |
| Kluge *et al.* (2015)[4] | Two-step collagenase perfusion. | **Number of livers:** 190  **Weight:** 32.2 ± 1.3 g | Albumin and urea secretion.  Purity: not specified | **Mean initial yield:** 9.9 ± 1.4 x 10^6^  **Mean initial viability:** 76.1 %  (Average between PVE and non-PVE group) |
| Bartlett *et al.* (2014)[5] | Two step collagenase or Liberase perfusion | **Number of livers:** 30  **Weight:** 88.7 g | Albumin and urea secretion.  Purity: not specified | **Median yield:** 0.28 × 10^6^  **Median viability:** 70 % |
| Lee *et al.* 2013[6] | Two-step collagenase perfusion | **Number of livers:** 648  **Weight:** 37 ± 29 g | IF: Albumin (n = 4).  Phase contrast imaging: typical morphology.  Purity: 94 ± 1 %  (n = 4) | **Mean yield:** 13 ± 11 x 10^6^  **Mean viability**: 77 ± 10 % |
| Gramignoli *et al.* (2012)[7] | Two-step collagenase perfusion. | **Number of livers:** 110  **Weight:** 201 ± 75 g | Phase contrast imaging: typical morphology.  Phase II conjugation activity.  CYP1A1/2 and CYP3A4 activity. Ammonia metabolism  Purity: not specified | **Mean yield:** 7.6 ± 5.5 x 10^6^  **Mean viability:** 80 % |
| Bhogal *et al.* (2011)[8] | Two-step collagenase perfusion | **Number of livers:** 104  **Weight:** 110 g | Albumin and urea secretion.  IF: CK18 staining  Purity: not specified | **Median yield:** 0.35 x 10^6^  **Median viability:** 40 % |

*Immunofluorescence (IF). Cytokeratin 18 (CK18).* *Tetramethylrhodamine, methyl ester* *(TMRM)*. *Portal vein embolisation (PVE).* *Studies that isolated human hepatocytes by referencing Seglen et al. or a protocol listed in Table S1 were excluded.*

### **Supplementary Methods**

**Supplementary** **Table 2.** Reagents required for PHH isolation and culture.

| **Reagent** | **Vendor** | **Catalogue #** |
| --- | --- | --- |
| Acridine Orange/Propidium Iodide Stain | Logos Biosystems | F20331 |
| Antibiotic-antimycotic | Gibco™, ThermoFisher Scientific | 15240-096 |
| Bovine albumin fraction V (7.5% solution) | Gibco™, ThermoFisher Scientific | 15260037 |
| B-27™ Supplement (50X), serum free | Gibco™, ThermoFisher Scientific | 17504001 |
| Calcium chloride (CaCl_2_) | Thermo Scientific | J63122.AD |
| Collagenase P | Roche | 11249002001 |
| Collagen Type I | STEMCELL Technologies Inc | 04902 |
| Dexamethasone | ThermoFisher Scientific | A13449 |
| Distilled water (sterile) | Gibco™, ThermoFisher Scientific | 15230188 |
| EGTA | Thermo scientific | J60767.AE |
| Fetal bovine serum (FBS) | Gibco™, ThermoFisher Scientific | A31605-01 |
| Hepatocyte Culture Medium (HCM) BulletKit^TM^ | Lonza | CC-3198 |
| Hank’s balanced salt solution (HBSS) (without magnesium and calcium) | Gibco™, ThermoFisher Scientific | 14170138 |
| Hank’s balanced salt solution (HBSS) (with magnesium, calcium, no phenol red) | Gibco™, ThermoFisher Scientific | 14025092 |
| Human recombinant EGF, ACF | STEMCELL Technologies Inc | 78136.1 |
| Human Recombinant bFGF, ACF | STEMCELL Technologies Inc | 78134.1 |
| Periodic Acid Schiff (PAS) stain kit | Abcam | ab150680 |
| PBS | Gibco™, ThermoFisher Scientific | 10010023 |
| Penicillin-Streptomycin (5,000 U/mL) (pen-strep) | Gibco™, ThermoFisher Scientific | 15070063 |
| Propidium Iodide (PI) cell counting/viability dye | Logos Biosystems | F23001 |
| Red blood cell (RBC) lysis solution | Sigma Aldrich | R7757 |
| Tissue storage solution | Miiltenyi Biotec | 130-100-008 |
| William’s E media (WME) | Gibco™, ThermoFisher Scientific | 3251020 |
| Y-27632 dihydrochloride | TOCRIS, Biotechne | 1254 |

**Supplementary** **Table 3.** Materials required for PHH isolation and culture.

| **Material/equipment** | **Vendor** | **Catalogue #** |
| --- | --- | --- |
| Biological safety cabinet (BSC) | ESCO Lifesciences group | AC2-2E8 |
| Blades | ASTRA | - |
| Cell culture plates - 96 and 24 well | Corning, Fisher Scientific, part of ThermoFisher Scientific | 10695951, 10380932 |
| Thermo Scientific™ Nunc™ Cell Culture/Petri Dishes | Fisher scientific, part of ThermoFisher Scientific | 0738581 |
| µ-Slide 8 Well | Ibidi | 80826 |
| LUNA-FL™ Dual Fluorescence Cell Counter | Logos Biosystems | L20001 |
| Cell strainer (100 µm) | STEMCELL Technologies Inc | 27270 |
| Centrifuge | Eppendorf | 5810R |
| Falcon tubes, 50mL | Corning | 352070 |
| Filter | MERCK | SLHPR33RB |
| GentleMACS C tubes | Miltenyi Biotec | 130-096-334 |
| GentleMACS Octo Dissociator with heaters  Or  Regular GentleMACS Dissociator | Miltenyi Biotec | 130-096-427  130-093-235 |
| Tumour dissociation kit, human | Miltenyi Biotec | 130-095-929 |
| Tweezers | Sigma-Aldrich | 930229 |
| Human waste disposal bags | Stericycle | Yellow waste bags |
| Incubator | Eppendorf | CellXpert C170i |
| MACSmix Tube Rotator | Miltenyi Biotec | 130-090-753 |
| PhotonSlide ™ | Labtech | L12004 |
| Scalpel | Swann-Morton | 0508 |
| Specimen tubs, container 250 mL | Stardest | 75.9922.534 |
| Fisherbrand™ Plastic PP Syringes, Luer Lock | Fisher scientific, part of ThermoFisher Scientific | 12941031 |
| Syringe filter (33 mm diameter, 0.45 µm pore size hydrophilic, Polyethersulfone (PES) membrane | Merck | SLHPR33RB |
| Vibratome | Leica | Leica VT1200 |
| Water bath (GLS aqua 12 Plus) | Lab exchange | 42455 |

**Supplementary** **Table 4.** Buffers and media.

| **Buffer/media** | **temperature** | **Reagents** | **Purpose** |
| --- | --- | --- | --- |
| EGTA buffer | 37 °C | HBSS (without magnesium and calcium), 0.5 mM EGTA, 0.5% fatty acid free bovine albumin fraction V | Disrupt epithelial cell intercellular junctions |
| Digestion buffer | 37 °C | HBSS (without magnesium and calcium), 1 mg/mL collagenase P, 0.5% fatty acid-free bovine albumin, 10 mM CaCl_2_.  The collagenase P media is filtered using a 0.45 µm Millipore syringe filter (SLHPR33RB) before use | Digest the tissue ECM and generate single cell suspension.  *Note*: Ensure sufficient volumes for additional digestion if required. |
| Cell plating media | Room temperature (RT) (Store volume for later at 4°C, warm to 37°C before adding to cells) | RPMI, Y-27632 dihydrochloride (10 µM), dexamethasone (100 nM), EGF (10 ng/mL), bFGF (20ng/mL), 1X antibiotic-anti-mycotic, 2% B27 supplement and 10% FBS. | Plating cells and changing cells after initial 4 h incubation |
| Vibratome cutting media | 4°C | HBSS (with magnesium, calcium, no phenol red), pen-strep  *Note:* HBSS without phenol red is required for the cutting step, but phenol red can be present in other HBSS steps. | Added to vibratome chamber. |
| Storage media for cut liver slices | 4°C | WME, pen-strep | container with cut tissue slices kept on ice. |
| HBSS | 4°C | HBSS (without magnesium and calcium) | Wash after cutting and removing blood vessels. |
| HBSS | RT | HBSS (without magnesium and calcium) | Wash after EGTA step. |
| WME | RT | WME | Initial wash after digestion step |
| WME | 4°C | WME | additional washing steps |
| PBS | 4°C | PBS | Neutralising RBC lysis buffer |
| Culture media | 37 °C | HCM | Culturing cells |

*Note:* Volumes required will depend on the size of the tissue dissociating.

**Supplementary** **Table 5**. Volumes of collagen solution are used to coat plates.

| **Plate format** | **Volume of collagen coating** |
| --- | --- |
| 12 well | 1 mL |
| 24 well | 500 µl |
| 96-well | 50 µl |

**Supplementary** **Table 6**. Primary and secondary antibodies and isotypes required for immunofluorescent staining.

| **Antibody** | **Species** | **Dilution** | **Vendor** | **Catalogue #** | **Lot #** | **Marker** |
| --- | --- | --- | --- | --- | --- | --- |
| Alexa Fluor® 488 anti-cytokeratin 8.  Monoclonal | Rabbit | 1:100 | Abcam | ab192467 | 10046583 | Hepatocytes. Cholangiocytes |
| Alexa Fluor® 555 anti-cytokeratin 19.  Monoclonal | Rabbit | 1:200 | Abcam | ab203444 | 1009321-11 | Cholangiocytes |
| Unconjugated anti-albumin.  Monoclonal | Mouse | 1:100 | Bio-techne | MAB1455 | 0000152751 | Hepatocytes |
| Unconjugated anti-CYP3A4.  Monoclonal | Mouse | 1:200 | ThermoFisher | MA5-17064 | WD3195083A | Hepatocytes |
| Alexa Fluor® 647 anti-vimentin.  Monoclonal | Mouse | 1:200 | Abcam | ab195878 | - | Mesenchymal cells |
| DAPI | - | 1:500 | Abcam | ab228549 | - | Nuclei |
| Purified Mouse IgG2b, κ  Monoclonal | Mouse | 1:25 | Biolegend | 400347 | - | Isotype |
| Alexa Fluor® 488  IgG, monoclonal isotype control.  Monoclonal | Rabbit | 1:100 | Abcam | ab199091 | GR3422308-3 | Isotype |
| Anti-mouse IgG secondary, Alexa Fluor® 555 | Goat | 1:500 | Invitrogen | A21424 | 2139320 | secondary |
| LipidSpot488 | - | 1:1000 | Biotum | 70065-T | - | Lipid dye |

Data not known indicated by (-).

**Supplementary** **Table 7.** Reagents and materials required for immunofluorescent staining.

| **Reagent** | **Vendor** | **Catalogue #** |
| --- | --- | --- |
| 4% formaldehyde | Cell signalling technology | 4776 |
| Triton X-100 | SIGMA | T8787-100ML |
| Tween 20 | SIGMA | P7949-100ML |
| PBS | Gibco™, ThermoFisher Scientific | 10010023 |
| BSA solution | SIGMA | A1595-50ML |
| Normal goat serum | ThermoFisher Scientific | PCN5000 |
| Cytovista antibody dilution buffer | Thermofisher scientific | V11305 |
| Mounting media | Ibidi | 5001 |

**Supplementary** **Table 8.** Reagents, antibodies and isotype for flow cytometry.

| **Reagent** | **Vendor** | **Catalogue #** | **Lot #** | **Dilution** |
| --- | --- | --- | --- | --- |
| LIVE/DEAD™ Fixable Blue Dead Cell Stain | ThermoFisher | L34961 | - | - |
| 4% Formaldehyde | Cell signalling | 47746 | - | - |
| Flow cytometry buffer | Miltenyi Biotec | 130-091-221 | - | - |
| 10X saponin permeating solution | ThermoFisher Scientific | J63209.AK | - | 10X diluted to 1X in 4°C sterile distilled water |
| Alexa Fluor® 488 anti-cytokeratin 8 antibody. (5mg/mL) | Abcam | ab192467 | 10046583 | 1:250 |
| Alexa Fluor® 488 rabbit IgG, monoclonal isotype control. (5mg/mL) | Abcam | ab199091 | GR3422308-3 | 1:250 |

### **Multiplex Immunofluorescence Imaging of Vibratome-Cut Liver Slices**

Multiplexed immunofluorescence imaging was performed using the Cell DIVE platform (Leica Microsystems, 2500HS) on vibratome-cut liver slices. Tissue sections were mounted onto Superfrost Gold™ slides (Thermo Fisher), which are essential for minimizing tissue loss during iterative staining and imaging cycles. After deparaffinization and a two-step antigen retrieval process, slides were permeabilized with 0.05% Triton X-100 in PBS for 5 minutes and subsequently blocked with 1% BSA, 1% normal goat serum, and normal donkey serum in 0.05% Tween-20 for 30 minutes at room temperature.

Sections were first stained with DAPI and imaged across all channels to capture tissue-specific autofluorescence (AF). This was followed by iterative rounds of antibody staining, each consisting of up to two markers plus DAPI per cycle. Each cycle included primary and secondary antibody incubation (or direct-conjugated antibodies), imaging, and fluorophore inactivation. The following primary antibodies were used: anti-pan-keratin (Type I) [E6S1S] (Cell Signaling Technology, #83957), anti-ATPase alpha 1 subunit (Abcam, ab274883), and anti-CD31 [EPR17259] (Abcam, ab182981).

Between imaging cycles, fluorophore bleaching was performed according to the Cell DIVE protocol: three consecutive 15-minute bleaching steps to fully inactivate prior fluorophore signals and prevent spectral overlap. After the final staining cycle, slides were washed and mounted using antifade mounting medium. Image acquisition was carried out using the Cell DIVE high-throughput system with sequential dye cycling. Image alignment and downstream processing were performed using the Cell DIVE image analysis pipeline.

### **Supplementary Figures**

**Supplementary Fig. 1. Vibratome-cut liver slices maintain structural and cellular integrity after 5 days in *ex vivo* culture**


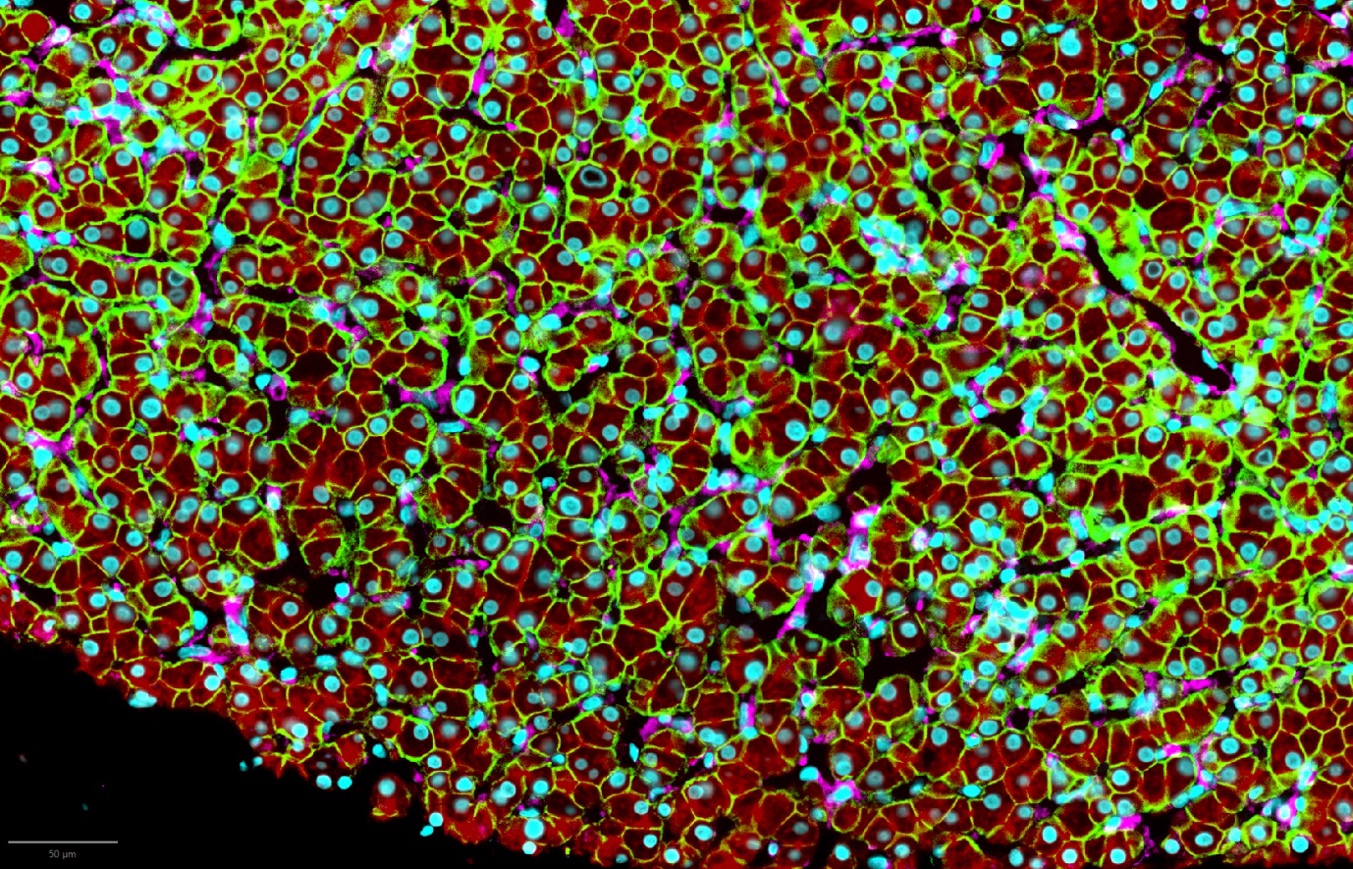


50 µm

DAPI/Pan Keratin/ATPase/CD31

Representative immunofluorescence image of a vibratome-sliced liver section stained for DAPI (cyan), pan-keratin (red, hepatocytes and bile ducts), ATPase (green, biliary canaliculi between hepatocytes), and CD31 (magenta, endothelial cells). The ATPase signal highlights canalicular membranes, reflecting preserved hepatocyte polarity and tissue architecture after slicing and culture. Scale bar = 50 μm.

**Supplementary Fig. 2 Protocol 4 yields more viable PHH than protocol 1**

Brightfield and immunofluorescent images of cells isolated from a fibrotic sample using **(a)** protocol 1 (Green) and **(b)** protocol 4 (Final), n = 1. Scale bar: Brightfield images = 25 μm, Immunofluorescent images = 100 μm. **(c)** Quantification of albumin-, CK8-, CK19- and vimentin-positive cells isolated using protocol 1 (Green) and protocol 4 (final).

**
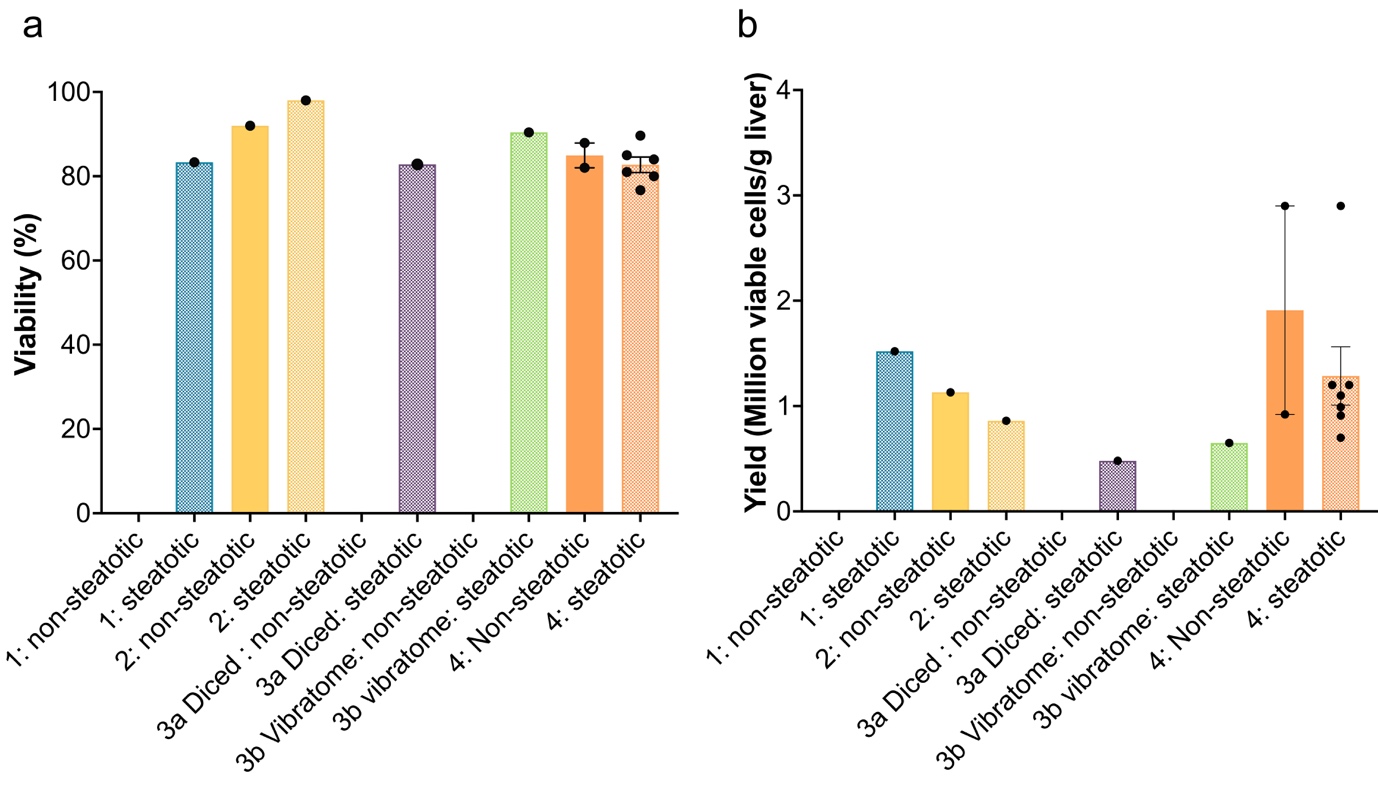
 Supplementary Fig. 3. The yield and viability of PHH isolated from steatotic and non-steatotic liver samples using each protocol**

Cell viabilities **(a)** and yields **(b)** of PHH from steatotic (n = 11) and non-steatotic (n = 3) liver specimens.

**Supplementary Fig. 4 Prior adjuvant chemotherapy does not affect viable hepatocyte yield**

Hepatocyte yields **(a)**, purities **(b)** and viabilities **(c)** from specimens obtained from patients with prior chemotherapy exposure (n = 7) and chemotherapy-naïve patients (n = 1). The fibrotic sample was excluded from the chemotherapy comparison, as fibrosis is known to reduce yield and viability [9].

**References**

1. Green, C.J., et al., *The isolation of primary hepatocytes from human tissue: optimising the use of small non-encapsulated liver resection surplus.* Cell and Tissue Banking, 2017. **18**(4): p. 597-604.

2. Pfeiffer, E., et al., *Featured Article: Isolation, characterization, and cultivation of human hepatocytes and non-parenchymal liver cells.* Exp Biol Med (Maywood), 2015. **240**(5): p. 645-56.

3. Kegel, V., et al., *Protocol for Isolation of Primary Human Hepatocytes and Corresponding Major Populations of Non-parenchymal Liver Cells.* J Vis Exp, 2016(109): p. e53069.

4. Kluge, M., et al., *Human Hepatocyte Isolation: Does Portal Vein Embolization Affect the Outcome?* Tissue Engineering Part C: Methods, 2015. **22**(1): p. 38-48.

5. Bartlett, D.C., et al., *Combined use of N-acetylcysteine and Liberase improves the viability and metabolic function of human hepatocytes isolated from human liver.* Cytotherapy, 2014. **16**(6): p. 800-9.

6. Lee, S.M., et al., *Isolation of human hepatocytes by a two-step collagenase perfusion procedure.* J Vis Exp, 2013(79).

7. Gramignoli, R., et al., *Development and Application of Purified Tissue Dissociation Enzyme Mixtures for Human Hepatocyte Isolation.* Cell Transplantation, 2012. **21**(6): p. 1245-1260.

8. Bhogal, R.H., et al., *Isolation of Primary Human Hepatocytes from Normal and Diseased Liver Tissue: A One Hundred Liver Experience.* PLOS ONE, 2011. **6**(3): p. e18222.

9. Lee, S.M., et al., *An algorithm that predicts the viability and the yield of human hepatocytes isolated from remnant liver pieces obtained from liver resections.* PLoS One, 2014. **9**(10): p. e107567.
